# Low‐Fluence Q‐Switch 1064 Nm Laser Combined With Oral Tranexamic Acid: A Quicker Treatment for Laser‐Induced Postinflammatory Hyperpigmentation

**DOI:** 10.1111/jocd.70018

**Published:** 2025-02-10

**Authors:** Wei Feng, Lichang Yang

**Affiliations:** ^1^ Center for Plastic and Reconstructive Surgery, Department of Plastic and Reconstructive Surgery Zhejiang Provincial People's Hospital (Affiliated People's Hospital, Hangzhou Medical College) Hangzhou China; ^2^ Department of Breast Oncoplastic Surgery, Hunan Cancer Hospital and the Affliated Cancer Hospital of Xiangya School of Medicine Central South University Changsha China

**Keywords:** laser‐induced PIH, Q‐switch 1064 nm laser, STROBE guidelines, tranexamic acid

## Abstract

**Background and Purpose:**

Laser‐induced post‐inflammatory hyperpigmentation (PIH)is a common adverse reaction in Asian individuals. Dark skin and incorrect laser parameters are common causes, but PIH is often unexpected for patients. Obvious hyperpigmentation can lead to an ugly appearance and severe anxiety. Therefore, a fast and effective treatment for laser‐induced PIH is necessary. In this article, we attempted to demonstrate that a low‐fluence Q‐switch (QS) 1064 nm laser combined with oral tranexamic acid (TXA) is a quick and safe method for the clearance of laser‐induced PIH.

**Materials and Methods:**

A retrospective cohort study, adhering to the Strengthening the Reporting of Observational Studies in Epidemiology (STROBE) guidelines, spanning 2 years (2021–2023) was conducted on 23 patients aged between 29 and 58 years. These patients were diagnosed with laser‐induced PIH for < 3 months. The treatment regimen was a low‐fluence QS1064 nm laser combined with oral TXA, oral TXA was first taken, and a low‐fluence QS1064 nm laser was used for at least 1 month after the last laser was applied. Three to six laser treatments were subsequently applied. After treatment, the pigment color and patient satisfaction were assessed to evaluate the effectiveness of the treatment options.

**Results:**

Twenty patients were enrolled in the study, the average laser treatments were 4.3 ± 0.865 times. The Melasma Area and Severity Index (MASI) score decreased from 9.325 ± 3.38 before treatment to 5.97 ± 2.37. After the first treatment, the MASI score decreased by approximately 40%. Two months after the last treatment, the MASI score decreased to 0.93 ± 1.06, and there was a statistically significant difference in the MASI score before and after treatment (*p* < 0.05). Patient satisfaction scores revealed that 95% of patients were highly or strongly satisfied with the decrease in the intensity of the PIH color, with a moderate response rate of 1 (5%). The patients thought that the pigment removal speed was 75% (15) very fast or fast, 20% (4) moderate, and 5% (1) slow.

**Conclusion:**

The results of this study demonstrated the quick and safe removal of laser‐induced PIH following treatment with low‐fluence QS1064 nm laser combined with oral TXA. Providing such a protocol is indeed one of the primary objectives of this article.

## 1 Introduction

Post‐inflammatory hyperpigmentation (PIH) is a skin hyperpigmentation secondary to an inflammatory response in the skin. Then, melanocytes are stimulated, leading to excessive production and deposition of melanin [1]. The most common PIH in plastic surgery is mainly caused by trauma and laser irradiation. Pigment generally appears very quickly in these patients, often at a maximum of clinical PIH within 4 weeks after injury [2, 3], and darker skin often appears to be very visible. Laser‐induced PIH is a common adverse reaction for Asian dark‐skinned people [4], but it is often unexpected to patients. Obvious hyperpigmentation in the skin often leads to an ugly appearance and severe anxiety for patients [5]. Although it gradually fades for approximately half to 1 year, PIH may significantly negatively affect the patient's life. Therefore, fast and effective treatment for laser‐induced PIH is necessary.

Compared with PIH caused by Ultraviolet B (UVB) or drugs, laser‐induced PIH is regulated by more processes involving fibroblasts, keratinocytes, and melanocytes. In addition, laser treatment may cause direct damage to the deeper layers of the skin, making the treatment of laser‐induced hypertension more difficult [2]. Currently, the use of a Q‐switch (QS) 1064 nm laser and tranexamic acid (TXA) are common methods for treating PIH [1, 6, 7]. Many studies have reported the safety and effectiveness of this combined treatment [8, 9]. Currently, we are attempting to develop a quick and safe method for the removal of laser‐induced PIH, which is very important for relieving patients' anxiety and improving their lives.

## 2 Materials and Methods

### 2.1 Study Design

A retrospective cohort study, adhering to the Strengthening the Reporting of Observational Studies in Epidemiology (STROBE) guidelines and spanning 2 years (2021–2023), was conducted on 20 patients. The therapeutic effects of laser‐induced PIH were compared by assessing the results of pigment changes before and after treatment. The primary outcome was the improvement in PIH color, and the secondary outcomes included patient satisfaction scores. In addition, adverse events were documented.

The study was conducted at the Plastic Surgery Department of Zhejiang Province Hospital and was approved by the Institutional Review Board Ethics Committee (approval no. 202406140939000059380). Written informed consent was obtained from each patient before enrollment.

### 2.2 Inclusion and Exclusion Criteria

Inclusion criteria: The study included 23 patients diagnosed with laser‐induced PIH for < 3 months, who showed no reduction in pigmentation or a trend towards reduction, and were therefore seeking quicker removal. Their ages ranged from 29 to 58 years with Fitzpatrick III or V.

The exclusion criteria included patients who had no active skin lesions in the treatment area; no underlying skin disease; a history of photosensitivity, abnormal scars, poor wound healing, oral contraceptives, or hormone replacement therapy in the previous month; a history of post‐inflammatory hyperpigmentation susceptibility; a history and type of coagulopathy, such as deep‐vein thrombosis, stroke, pulmonary thromboembolism, disseminated intravascular coagulation, or cancer; the use of drugs that interact with TXA or other anticoagulants or hormonal drugs, such as birth control pills; current pregnancy or lactation; or a planned pregnancy during treatment.

### 2.3 Treatment

When patients were diagnosed with laser‐induced PIH, oral TXA at a dose of 250 mg twice per day was first administered. Low‐fluence QS1064 nm laser treatment is scheduled to start at least 1 month after the last laser, and before QS1064 nm laser treatment, the condition of the facial skin needs to be evaluated. The patient was notified of any itching, rash, abnormal redness, or other unhealthy skin problems.

**FIGURE 1:**
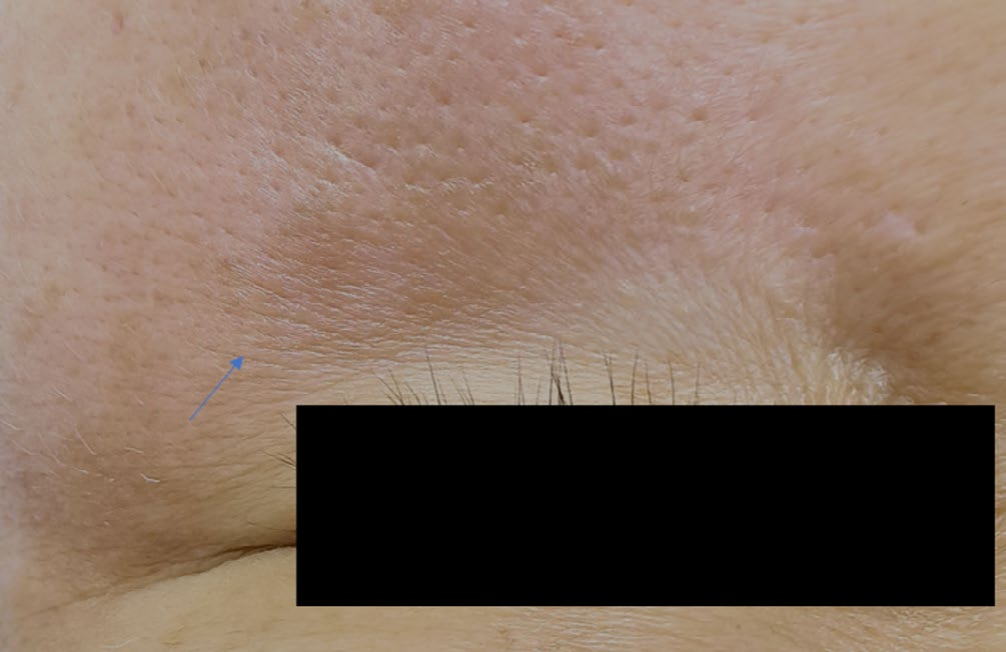
A slight epidermal oedema immediately after low‐fluence QS1064 nm laser treatment.

A low‐fluence QS1064 nm laser is applied 3–6 times, with intervals of one to one and a half months. The treatment parameters were as follows: 1064 nm wavelength (Medlite C6, HOYA ConBio, Fermont, CA), 6 mm spot size, 3.0–3.2 J/cm^2^, 10 Hz, and scanning at a constant speed perpendicular to the skin. The treatment area included the PIH and surrounding normal skin. Three to five passes until slight epidermal edema occurs (Figure 1) and obvious redness occurs in the pigmented area, and the normal skin shows mild redness.

Treatment should be stopped once the pigment lesion disappears. For laser‐induced PIH after melasma IPL treatment, a mild brown pigment or pigment no darker than the melasma pigment and a pigment area less than or equal to the melasma pigment before treatment can be considered treatment termination. Long‐term follow‐up is necessary to determine whether pigmentation will recur after stopping treatment. Patients are required to maintain strict sun protection after treatment, and only moisturizing skin care products are allowed.

### 2.4 Outcome Evaluation

The primary outcome was the improvement of PIH color, which was evaluated by Melasma Area and Severity Index (MASI) score. Standardized photographs were taken from the front and sides of both cheeks using a clinical imaging system (VISIA‐CR; Canfield Scientific, Parsippany, NJ). Images were taken at baseline, before each treatment, and 2 months after the last treatment. Two dermatologists who were blinded to the treatments performed objective clinical assessments of the PIH by MASI score before and 1 month, 3 months, 2 months after the last treatment, respectively. About half a year after the last laser treatment, we conducted a telephone follow‐up, as not all patients were willing to return to the hospital for a follow‐up visit.

Secondary outcome included patient satisfaction score, to evaluate overall patient satisfaction with the treatment, patients were asked to evaluate the extent of pigment removal and the removal speed of the PIH. Pigment improvement: slightly improved = 1%–25% clinical improvement, moderate improved = 26%–50% clinical improvement, much improved = 51%–75% clinical improvement, very much improved ≥ 75% clinical improvement; pigment removal speed: very fast, fast, fair, slow, very slow.

### 2.5 Statistical Analysis

Numerical and ordinal efficacy results are summarized using descriptive measures such as the mean, standard deviation, mean change from the MASI score, and the percentage of patient satisfaction score. The Student's *t*‐test was used to compare MASI scores at baseline, 1 month, 3 months, and 6 months. A 5% significance level was used in all analyses. Patient satisfaction scores were assessed using the percentage of responses. The GraphPad Prism 5 (GraphPad Software Inc., La Jolla, USA) was used for the analyses.

## 3 Results

### 3.1 Patient Characteristics

We screened 23 patients and included 20. One patient could not be included because she met one of the exclusion criteria. Two patients dropped out during treatment. Thus, data analyses were based on 20 patients.

Among the 20 patients, PIH occurred in less than 3 months, seven were caused by QS or picosecond laser treatment, seven were caused by fractional laser treatment, and six were caused by intense pulsed light (IPL) treatment. In fact, many of our PIHs caused facial hyperpigmentation, primarily due to melasma irritation, which often further aggravated patients' anxiety and dissatisfaction with PIH.

### 3.2 Treatment Results: MASI Score

**FIGURE 2:**
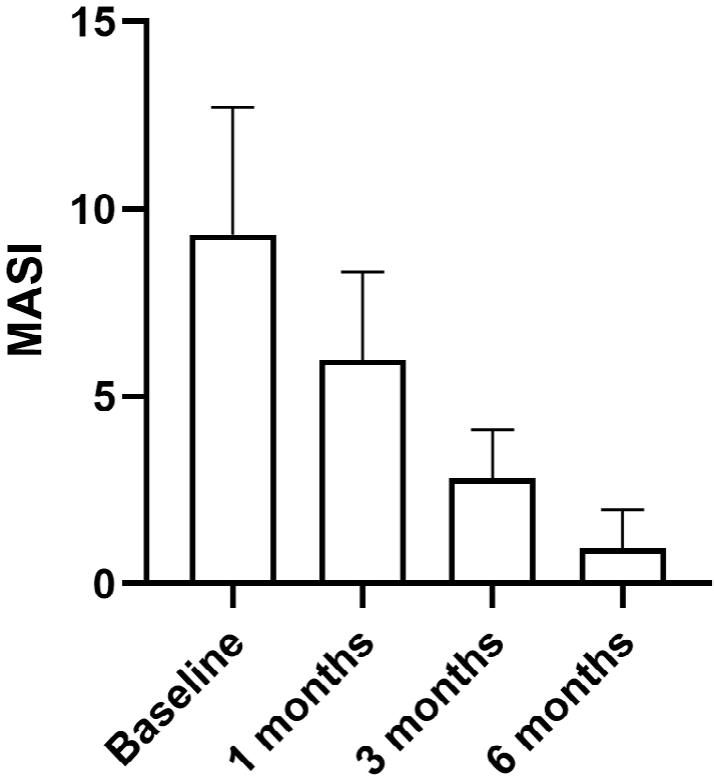
The MASI score before and 1 month, 3 months, 6 months after the treatment, respectively, there was a statistically significant difference in the MASI score before and after treatment (*p* < 0.05).

Among the 20 patients who underwent 3–6 treatments, the average treatment time was 4.3% ± 0.865%, the MASI score decreased from 9.325 ± 3.38 before treatment to 5.97 ± 2.37 after the first treatment, the MASI score decreased by approximately 40%, and 2 months after the last treatment, the MASI score decreased to 0.93 ± 1.06, the MASI score reduced by approximately 90%. There was a statistically significant difference in the MASI score before and after treatment (*p* < 0.05) (Figure 2). The detailed MASI scores are listed in Tables 1 and 2. During the telephone follow‐up of 6 months after the final treatment, no patients complained of a recurrence of PIH.

**TABLE 1:** Baseline demographics of the patients.

Characteristic	*N* = 20
Age, median (range)	41 (31–58)
Sex	
Male	1 (5%)
Female	19 (95%)
Number of treatments	
Session, mean (SD)	4.3 (0.865)
MASI score	
Initial scoring, mean (SD)	9.325 (3.38)
1 month after mean (SD)	5.97 (2.37)
3 months after mean (SD)	2.82 (1.29)
6 months after mean (SD)	0.93 (1.06)

**TABLE 2:** Treatment data.

Patient no.	Sex	Age (years)	Cause of PIH	Initial MASI score	MASI score after 1 month	MASI score after 3 months	MASI score 1 month after last treatment
1	Female	44	1	13.2	8.4	3	0.9
2	Female	43	2	4.8	2.4	2.4	0
3	Female	39	1	10.8	6	2.4	1.2
4	Female	35	3	7.2	6	3.6	0
5	Female	36	3	9	6	2.4	0.9
6	Female	37	3	7.2	5.4	2.4	0
7	Male	41	1	3	1.8	1.2	0
8	Female	33	1	15	12	4.8	2.1
9	Female	46	2	5.4	1.8	1.2	0
10	Female	32	2	7.5	4.5	2.4	1.2
11	Female	52	1	10.5	6.3	4.8	3.6
12	Female	31	2	7.2	4.8	3.6	1.2
13	Female	35	3	9	6	0	0
14	Female	49	1	13.2	8.4	3.6	2.4
15	Female	29	2	7.6	6	4	0
16	Female	42	2	9.9	6	3.6	2.4
17	Female	49	3	9	6	1.2	0
18	Female	43	2	9.9	6.3	3.6	1.2
19	Female	58	3	10.5	8.4	4.2	1.5
20	Female	49	2	16.6	6.8	2	0

*Note:* The cause of PIH, 1 for IPL treatment, 2 for QS or picosecond laser, 3 for fractional laser.

Further analysis of the MASI score and treatment time revealed a positive correlation, with a higher MASI score for PIH, indicating that there was more severe pigmentation, which indicates a more severe appearance. A decrease in the MASI score of 40% after the first treatment indicates obvious progress in pigment removal. A continued decline of MASI score in 1 month, 3 months, and half a year indicates that the treatment protocol is working ceaselessly. A total of 95% of patients achieved more than 75% improvement after the last treatment, 45% of patients achieved 100% improvement, and the patients appraised the speed of PIH removal (Figure 4). In addition, we found that in the early stage of treatment, within the first 3 months, PIH improvement first manifested as a decrease in pigment color and then an improvement in pigment uniformity, and a smaller pigment area was the last to appear.

### 3.3 Treatment Results: Patient Satisfaction Score

The patient satisfaction score is shown in Table 3. In total, 95% of the patients were highly or strongly satisfied with the intensity of the PIH color, 1 (5%) had moderate responses, 0 (0%) had poor responses, 75% (15) of the patients thought that the pigment removal speed was very fast or fast, 20% (4) of the patients thought that the speed was moderate, and one patient thought that the method for removing pigments was slow. The detailed treatment data are listed in Table 3.

**TABLE 3:** Patient satisfaction score (*n* = 20).

Pigment improvement	No. of patients
Worsened/unchanged	0 (0%)
Slightly improved	0 (0%)
Moderately improved	1 (5%)
Much improved	3 (15%)
Very much improved	16 (80%)
Pigment removal speed	
Very fast	6 (30%)
Fast	9 (45%)
Moderate	4 (20%)
Slow	1 (5%)
Very slow	0 (0%)

*Note:* **Patient report 1** (Figure 3). A 44‐year‐old female with IPL‐induced PIH in her cheek was very anxious about having a dark brown pigment and very willing to receive treatment involving four sessions of a low‐fluence QS 1064 nm laser combined with oral TXA. (a, e) Photo was taken before treatment; (b, f) photo after 1 month of treatment; (c, g) after 3 months; (d, h) half a year after treatment. The PIH had almost disappeared, and the MASI decreased from 13.2 to 8.4 1 month later, 3 three months later, and 0.9 half a year later, respectively. **Patient report 2** (Figure 4). A 43‐year‐old female with picosecond laser‐induced PIH for the treatment of melasma and solar sunspots received five sessions of a low‐fluence QS 1064 nm laser combined with oral TXA. (a, f) Photos were taken before picosecond laser treatment; (b, g) PIH; (c, h) after 1 month of treatment; (d, i) after 3 months; (e, j) half a year after treatment. The MASI decreased from 9.9 to 6 a month later, 3.6 3 months later, and 2.4 half a year later, respectively.

**FIGURE 3:**
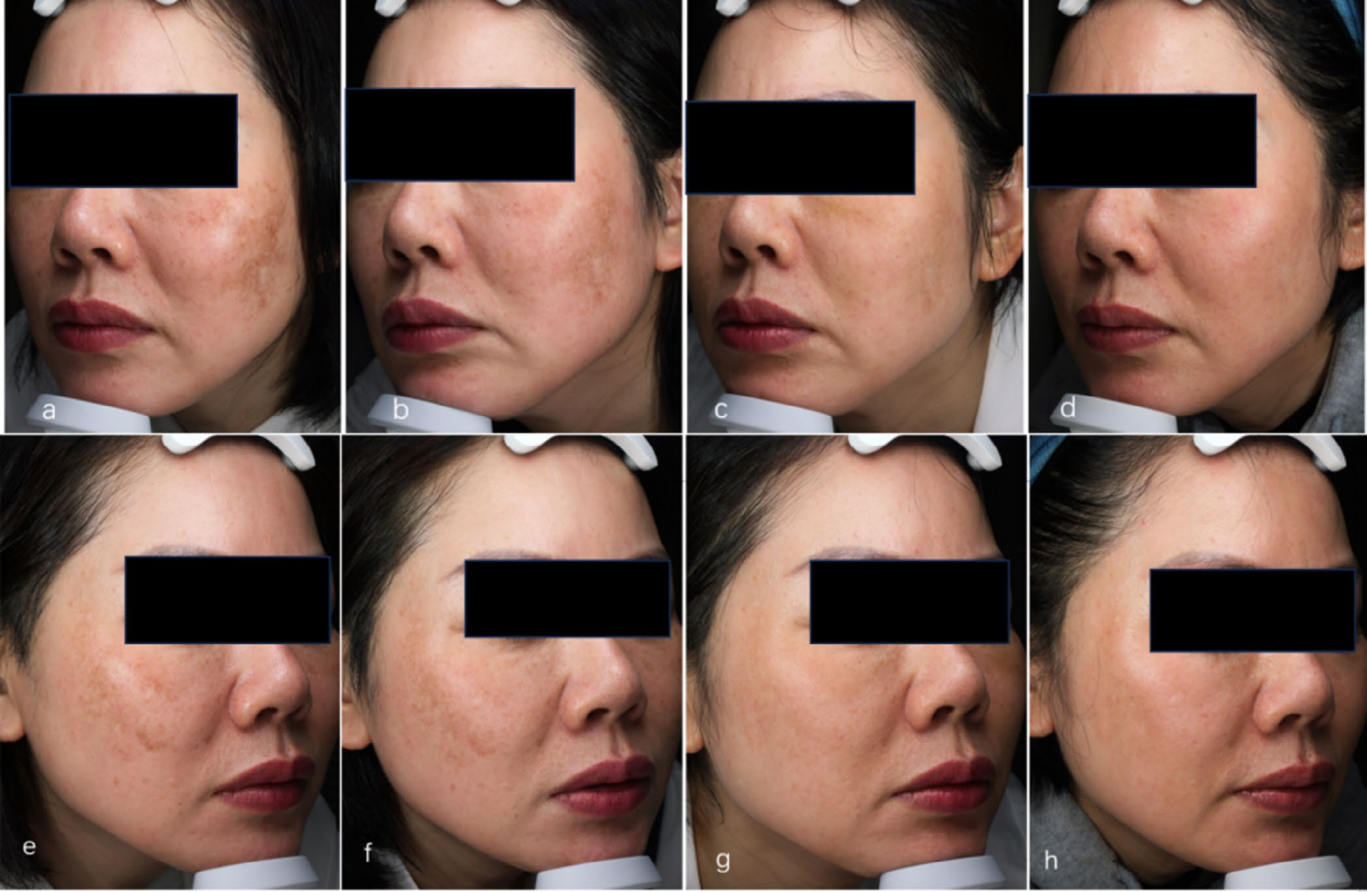
A 44‐year‐old female with IPL‐induced PIH in her cheek. Before (a, e), 1 month (b, f), 3 months (c, g), 6 months after treatment (d, h). The PIH had a significant improvement after treatment.

**FIGURE 4:**
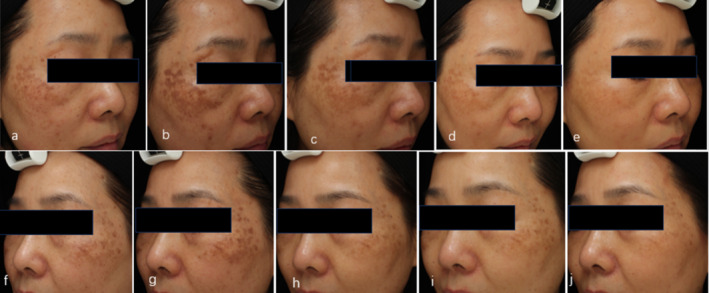
A 43‐year‐old female with picosecond laser‐induced PIH for the treatment of melasma and solar sunspots. Before (a, f); the PIH (b, g), 1 month (c, h), 3 months (d, i), 6 months after treatment (e, j). The PIH had a significant improvement after treatment.

### 3.4 Adverse Effects

One patient (5%) experienced nausea after oral TXA. Hypomenorrhea was reported in nine patients (45%), and the symptoms disappeared immediately after discontinuation of treatment. There was no incidence of vascular thrombosis during the treatment period. Two patients reported rash and itching the first day after laser treatment, but the symptoms disappeared after topical use of mometasone furoate cream for 1 day. There were no reports of worsened or hypopigmented pigmentation for any of the 20 patients.

## 4 Discussion

As Kristie described [10], when examining the causes of PIH, acne emerges as the primary inflammatory condition, while laser treatments are identified as the leading external cause. Laser therapy is a prevalent exogenous factor contributing to PIH, particularly in individuals with skin color [6], which has become more common due to the general use of cosmetic procedures in Asia [4]. The incidence has been reported to be 10%–47% in previous studies of Asian patients, which can cause major cosmetic concerns [2]. PIH occurs in 3%–28% of patients after IPL treatment for melasma. 10%–25% for QS lasers and 68%–92% for fractional CO_2_ lasers [2].

ZACHARY [11] summarized 494 cases of laser‐induced complications reported by the U.S. Food and Drug Administration between 2006 and 2011. The most common reason was user error by a health care provider (30%), followed by laser device malfunction (20%) and patient error (4%).

Compared with UVB‐ and laser‐induced PIH, the heat produced by inflammatory processes caused by laser treatment is more involved in the regulation of processes in fibroblasts, keratinocytes, and melanocytes. Skin inflammation caused by laser‐induced keratinocyte damage can produce inflammation. Mediators produce cytokines, chemokines, and other inflammatory mediators. The presence of these molecules leads to melanogenesis by increasing tyrosinase activity, ultimately leading to PIH due to laser irradiation. In addition, laser treatment may cause direct damage to the deeper layers of the skin, making the treatment of laser‐induced hypertension more difficult [2]. Long‐pulse lasers, such as fractional CO_2_ lasers and IPL, destroy melanin‐rich melanocytes and keratinocytes via photothermal effects, whereas short bursts of high‐energy QS lasers rupture melanosomes and melanin‐containing cells via both photothermal and photomechanical effects. Photomechanical actions affect surrounding superficial vessels and thereby produce inflammation through acoustic effects, while IPL and long‐pulse lasers, such as fractional CO_2_ lasers, cause inflammation through the laser heat effect. Although the precise pathogenesis of laser‐induced PIH remains unknown, inflammation is undoubtedly the mechanism underlying PIH development [12].

TXA is a drug commonly used to treat melasma [13] but has recently been used as a treatment for PIH [1]. The mechanism of TXA treatment for PIH is unknown. A previous study suggested that TA inhibits melanin synthesis in melanocytes by interfering with the interaction between melanocytes and keratinocytes through inhibition of the plasminogen/plasmin system [5].

Myoung [14] reported that TXA can reduce melanin synthesis by reducing tyrosinase protein expression in laser‐treated mouse and human keratinocytes, and he suggested that TXA may be an attractive candidate for the treatment of PIH [1]. Suthinee [3] attempted to use oral TXA to prevent pigmentation after the use of a QS532‐nm Nd:YAG laser for solar lentigines, and the results revealed that TXA was not effective at preventing pigmentation but could improve laser‐induced pigmentation beginning at the sixth week after laser treatment. Liang demonstrated that the oral administration of TXA is more effective than the intradermal injection [13], which is why we chose the oral administration of TXA because of its high rate of hypomenorrhea. It disappears immediately after stopping the drug.

Rutin is used at 1500 mg daily for 6 weeks for the treatment of PIH [3]. Young and HARUNOSU used 750 mg/d, which is higher than the dose we used [8, 15, 16]. There are currently no relevant studies that have confirmed that higher doses will have stronger effects. However, since our department has used 500 mg/day for the treatment of melasma for more than 10 years, the follow‐up results are safe, so we still chose 500 mg/day [17].

Shin [18] evaluated the effectiveness of oral TXA paired with low‐fluence QS1064nm laser versus QS1064nm laser alone for melasma treatment. After 2 months, a higher number of patients in the combination group achieved a score of grade 3 or higher; whereas, no patients in the laser‐only group received a grade 4 score. Ariya [19] compared the efficacy of QS1064 nm laser combined with the local use of 3% TA versus laser therapy alone for the treatment of melasma. The laser parameters were a fluence between 2.0 and 3.0 J/cm^2^ and a 6‐mm spot size. No significant decrease in MRSI was observed after 2 months of solo treatment. However, a significant decrease in MRSI was noted in the combined treatment group. Both of the above studies indicated that the combined therapy can lead to an early decrease in MRSI, which provides a theoretical basis for the combined treatment of laser‐induced PIH [10].

The low‐fluence QS1064 nm laser is the most commonly used laser to treat PIH on colored skin. Many previous studies have confirmed the effectiveness in treating pigmentation. Zawar reported that 78 patients (SPT IV–VI) with acne‐induced PIH resistant to topical therapy were successfully cleared by low‐fluence QS1064 nm laser treatment for six sessions at 2‐week intervals [20].

At present, most scholars still choose to use large‐spot and low‐energy treatment modes. Chen used a 1064‐mm spot‐size laser and an energy fluence of 1.6–1.8 J/cm^2^ combined with vitamin C for facial hyperpigmentation, and 91.3% of the patients showed an excellent or greater improvement in PIH [4]. Ghannam chose a 7–8‐mm spot size of QS1064 nm and an energy fluence of 1.4 J/cm^2^ and 5 Hz for five passes for the treatment of PIH; 3–12 sessions were needed after an interview of 2 weeks [21]. The endpoint tissue response to a low‐dose QS 1064 nm laser is mild redness, so more treatments and frequent intervals are often needed. Huang tested a higher‐energy QS 1064 nm laser (4.0–4.5 J/cm^2^, 4 mm spot size, 10 Hz, 2–5 times) for permanent PIH in 15 patients. The immediate intradermal bleeding point was considered the endpoint response. After at least four sessions, 1–2 months apart, 73% of patients achieved more than 50% improvement [22] and Chotied [23] reported a medium‐energy treatment of QS 1064 at 3.5–5 J/cm^2^, with a 5 mm spot and an interval of 1 month to treat Riel's melanosis. The endpoint reaction is subcutaneous bleeding or slight epidermal edema. None of those patients reported an aggravation of pigment color, and those who demonstrated an endpoint of subcutaneous bleeding and epidermal oedema were safe, which is consistent with what we clinically observed. According to our experience, when using a 6 mm spot size 1064 nm laser with a fluence of 3.2 J/cm^2^ to treat early‐stage pigmentation, a frequency of 10 Hz is too high for local small‐scale treatment for dark brown pigmentation, which might cause the pigment particles to fall off while reoccurring 1–2 weeks later, which is unsafe for PIH treatment. Therefore, we recommend using a frequency of 5 Hz. For large areas of the PIH, 10 Hz is a safe frequency. Because there may be dermal layer damage in laser‐induced PIH, when the pigment appears darker or more intense, 3–4 passes of treatment might cause an epidermal edema reaction, which is relatively safe. The treatment should be terminated, and skin cooling is needed.

The results of this study revealed a significant decrease in MASI scores in all patients after treatment with a QS 1064 nm laser combined with oral TXA. In particular, the PIH produced by fractional laser treatment for acne scars and QS/ps laser treatment for solar sunspots seems to fade more easily. For PIH caused by IPL and QS/ps laser treatment of melasma areas, a significant improvement in melasma was observed after treatment. Overall assessment revealed that patients were satisfied with the effect and speed of PIH improvement. The obvious reduction in pigment after the first treatment also relieved the anxiety of the patients.

## 5 Limitations

1. Like many other clinical reports on PIH, these studies have small sample sizes, are nonrandomized in design, and lack control groups. Therefore, larger, randomized controlled trials that investigate therapies for each skin type are warranted to optimize treatment strategies [21], and that is what we are going to do.
2. More sophisticated studies should be performed for the potential beneficial effects of QS1064 nm laser and TXA against the activity of melanocyte or melanophores in laser‐induced PIH.

## 6 Conclusion

As numerous studies have demonstrated, dark skin and improper laser parameters are prevalent causes of laser‐induced PIH [2, 4, 6]. Achieving optimal laser treatment outcomes while minimizing complications typically necessitates the accumulation of extensive clinical experience by physicians. For clinicians with limited experience, the availability of a reliable protocol is crucial for managing PIH in a safe and effective manner. Providing such a protocol is indeed one of the primary objectives of this article.

## Disclosure

All authors have read and approved the final manuscript. The authors alone are responsible for the content and writing of this article.

## Ethics Statement

The authors have nothing to report.

## Conflicts of Interest

The authors declare no conflicts of interest.

## Data Availability

The data that support the findings of this study are available from the corresponding author upon reasonable request.
